# Real-time laser ultrasonic monitoring of laser-induced thermal processes

**DOI:** 10.1038/s41598-022-13940-5

**Published:** 2022-06-14

**Authors:** Rosa E. Morales, Kathryn J. Harke, Joseph W. Tringe, David M. Stobbe, Todd W. Murray

**Affiliations:** 1 Department of Mechanical Engineering, University of Colorado, 1111 Engineering Drive, Boulder, CO 80309 USA; 2 Lawrence Livermore National Laboratory, Livermore, CA 94550 USA

**Keywords:** Mechanical engineering, Optical techniques, Acoustics, Characterization and analytical techniques

## Abstract

Intra- and inter-layer integrity of components fabricated with advanced manufacturing techniques, such as laser powder bed fusion, is dependent upon rapid heating, melting, and solidification processes. There is a need for new techniques to provide in situ feedback of these processes. Here a laser-based ultrasonic technique to probe thermal effects induced by a high-power continuous wave laser in titanium samples is described. Numerical simulations were performed to show that, for a spatially uniform heating beam, laser-induced surface acoustic waves are strongly influenced by surface heating conditions, are dispersive in the case of rapid heating, and that an abrupt velocity reduction happens upon the onset of surface melting. Furthermore, laser-based ultrasound experimental results which monitor the transient change of surface wave travel time associated with high power laser surface heating are provided. A pulsed laser is used to generate high frequency surface acoustic waves that propagate through the laser-heated region and are detected using a photorefractive crystal-based interferometer. Qualitative agreement is observed between theory and experiment with both showing a rapid reduction in the surface wave velocity at the onset of illumination and further decrease in surface wave velocity associated with melting. It is demonstrated that changes in the surface wave velocity can be used to track local heating and detect the onset of surface melting in real time.

## Introduction

Lasers have seen widespread use in materials processing and manufacturing applications including laser cutting, welding, ablation, drilling, surface texturing, and advanced manufacturing^1^. In these applications, laser energy is absorbed by a material leading to local heating, melting, and vaporization, and in situ control of these laser-induced processes is critical to ensure the integrity of the final product^2^. For example, in laser powder bed fusion an object is built in consecutive layers by laser melting of powder mechanically distributed over the build surface^3,4^. Intra- and inter-layer integrity is dependent upon the rapid heating, melting, and solidification processes during which defects and material discontinuities are likely to form^3–6^. If the heating laser power is too high for a given scan speed, it can lead to evaporation of the material from a molten pool, a subsequent recoil force, and collapse of the melt pool, resulting in porosity in the component. Alternatively, a low laser heating power can lead to an incomplete melting and solidification process and lack of fusion defects^7,8^. The optimal laser parameters, defined as those required to produce a part with the lowest number of processing defects, are difficult to determine a priori due to the complexity of the process and additional variables including powder quality and machine to machine variations in laser characteristics^9^.

The success of laser-based manufacturing techniques, such as powder bed fusion, hinges on the ability of the operator to set and control process variables such as laser power and speed^10,11^. Nondestructive evaluation can aid in this process and provide in situ feedback on the build quality such that process variables can be adjusted in real time^12^. Several nondestructive evaluation methods such as thermal imaging, optical imaging, and conventional ultrasonics have been employed to monitor laser powder bed fusion builds^9,12^. These techniques offer remote access to the high temperature laser-material interaction site. Thermal imaging can provide information regarding the surface temperature distribution, and optical imaging can be used to ascertain surface morphology changes^9^. Conventional acoustic emission and ultrasound can potentially provide additional data regarding the build process^13^. In the case of acoustic emission, the sound generated during the laser heating process is detected using a microphone or transducer and analyzed to infer information about the process^14^. Using signal processing techniques, including those employing machine learning, it is possible to categorize the laser-material interaction into different regimes^15^.

In addition, the physical, mechanical, optical, and thermal properties of materials are functions of temperature. It follows that the velocity of ultrasonic waves is also temperature-dependent with an increase in temperature generally leading to a softening of the material and lower longitudinal, shear, and surface acoustic wave velocities. Ultrasound thermography is a well-known technique used to determine the temperature of a uniformly heated sample or to map out temperature distributions below the surface^16^. Ultrasound thermography has attracted much attention in the medical ultrasonics community to continuously monitor and provide feedback to thermal treatment processes in biological tissue such as high intensity focused ultrasound therapy^16,17^. Some of the advantages of conventional ultrasonic techniques for process monitoring are that they are not limited to surface temperature determination and that the transducers can be placed remote from the process zone. For additive manufacturing, they do require physical access to the build surface, but the contact location can be somewhat removed from the high temperature environment. The dependence of bulk wave propagation on temperature, mechanical properties, and phase state of the material has, for example, been used to infer the temperature in the processing zone, and to predict the melt pool size based on monitoring bulk waves reflected and scattered from the melt pool^18,19^.

Laser-based ultrasonic techniques are well suited for the real-time monitoring and nondestructive evaluation of laser-induced thermal processes. The laser detection probe beam and laser source exciting the ultrasonic waves can be separated from the high temperature manufacturing process environment using optically transparent windows or other means. Laser ultrasonic techniques have been used to evaluate advanced manufacturing builds by using surface acoustic waves and bulk waves to detect surface and near subsurface defects *ex situ*^20–23^. Surface acoustic waves and bulk waves have also been used to evaluate material microstructure and grain size^24^, to infer the surface temperature in laser-induced thermal processes^22,25^, to predict internal temperature distributions based on waves propagating over multiple paths^26^, and to observe melting and solidification during crystal growth^27^. Finally, laser ultrasonic methods have been used for high temperature measurements of materials properties^28^ and for phase transformation studies in metals^29–31^.

In this work, laser-based ultrasound is used to monitor laser-induced heating and melting processes. Numerical simulations of a spatially-uniform heating beam show that laser-induced surface acoustic waves are strongly influenced by surface heating conditions. They are dispersive in the case of rapid heating where the thermally-induced mechanical property change is on the same spatial scale as the wavelength of the surface acoustic waves, and they show an abrupt velocity reduction upon the onset of surface melting. Furthermore, using a laser line source for surface acoustic wave excitation and a point interferometric detector, experimental results demonstrate the transient change of surface wave travel time associated with high power laser surface heating with a Gaussian beam placed between the source and receiver positions. In agreement with the numerical simulations, a deviation in the response is observed when the heating laser power is sufficient to cause local surface melting. The proposed technique may find effective application in the mapping of transient laser-induced thermal fields and melt zones, providing critical information for real-time process control.

## Background and theory

**Figure 1:**
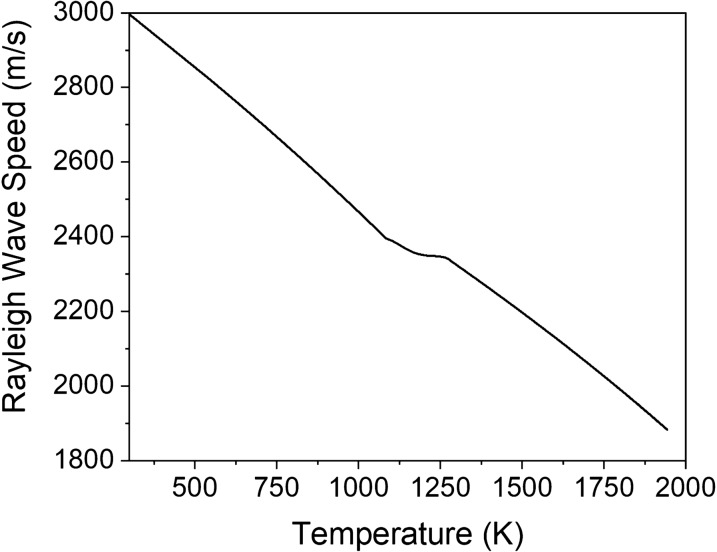
Rayleigh wave speed of Ti-6Al-4V as a function of temperature ranging from room temperature to the melting temperature of 1943 K.

The thermal and elastic properties of materials are dependent on temperature, with an increase in temperature generally resulting in a decrease in both material density and stiffness. A uniformly heated sample of a titanium alloy (Ti-6Al-4V) that is assumed to be homogeneous and isotropic is considered first. The temperature-dependent density, elastic modulus, and Poisson’s ratio are obtained from the software *JMatPro*^32^ and are used to determine the longitudinal, ${c}_{L}$, and shear, ${c}_{T}$, wave speeds as a function of temperature. These values are, in turn, used to calculate the Rayleigh wave speed using the characteristic equation^33^. The result is shown in Fig. 1 where the Rayleigh wave speed is seen to decrease from 3000 m/s at room temperature (293 K) to 1880 m/s at the melting temperature of 1943 K. The decrease is relatively monotonic outside of a small region between 1100 and 1275 K, the temperature range at which Ti-6Al-4V undergoes an hcp (α) → bcc (β) phase transformation^34^. In the case of uniform heating, the thermal field and elastic properties are not depth-dependent and the Rayleigh waves propagate without dispersion.

**Figure 2:**
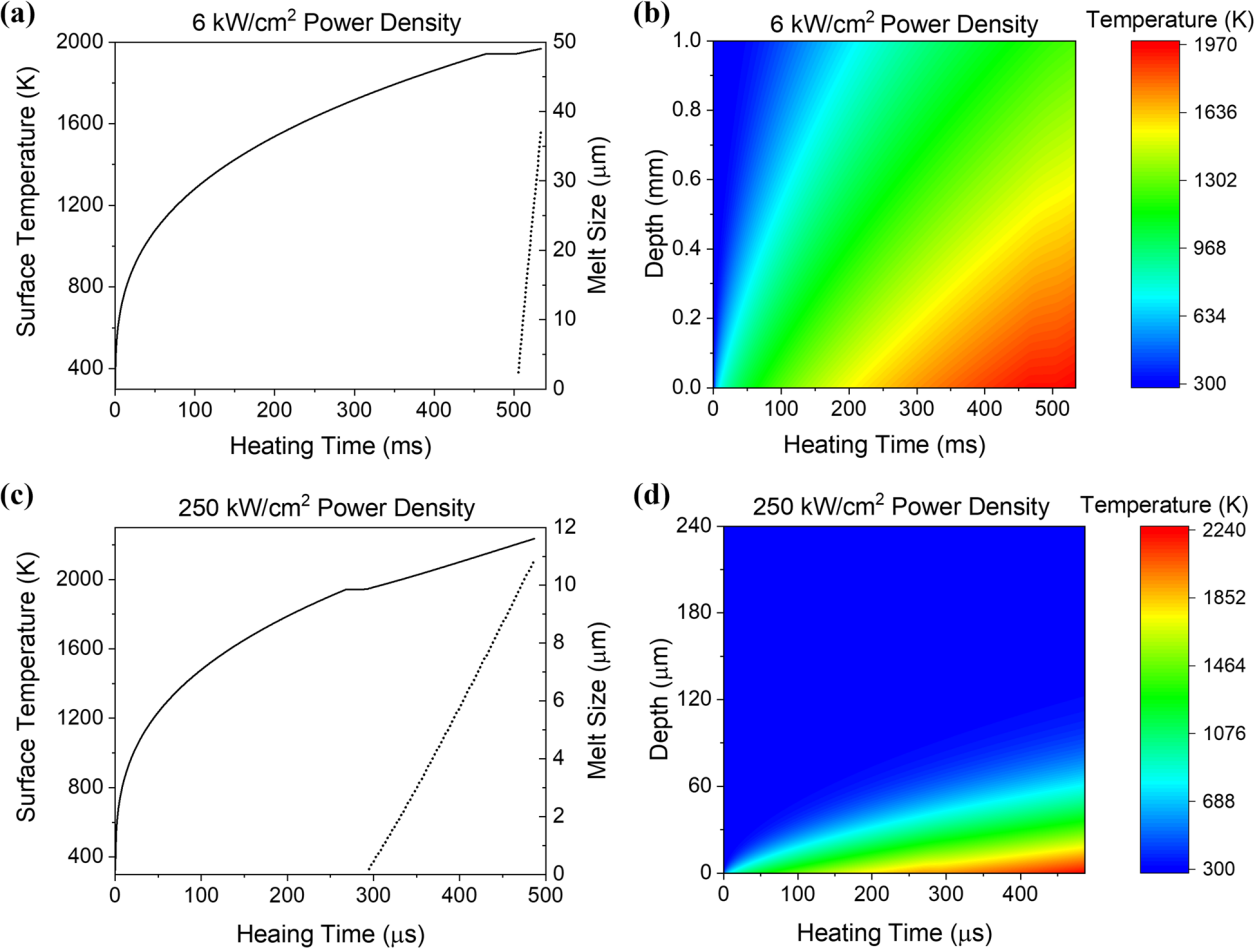
(**a**) Surface temperature (solid line) and melt size (dotted line) as a function of heating time for a 6 kW/cm^2^ laser power density incident on Ti-6Al-4V. (**b**) Calculated temperature field as a function of heating time and depth for the 6 kW/cm^2^ power density. (**c**,**d**) Results for a higher laser power density of 250 kW/cm^2^.

Rayleigh wave propagation becomes more complex in the case of transient heating, such as that produced by a high power laser source, as the thermal field and temperature-dependent elastic properties are functions of both time and space. Furthermore, the thermal properties are also temperature-dependent, and, at sufficiently high heating powers, the material will undergo a phase transformation resulting in surface melt. Here, the elastic displacement response generated by a nanosecond pulsed laser source incident upon a surface that is being heated by a spatially uniform continuous wave (CW) laser with a step-function time dependence is calculated. First, the one-dimensional temperature field produced by a CW laser is calculated using the implicit finite difference method presented by Singh and Narayan^35^. Temperature-dependent thermal properties and density are included for both the solid and liquid Ti-6Al-4V phases^36–39^. These properties are summarized in the Appendix. The model is used to determine the temperature as a function of time (*t*) at each depth (*d*) below the surface. Figure 2a shows the surface temperature (at *d* = 0) as a function of time where the heating laser is turned on at *t* = *0* and a heating laser power density of 6 kW/cm^2^. The surface temperature rises until it reaches the melting temperature of 1943 K at a time of 465 ms, where it briefly remains until the net heat absorbed exceeds the latent heat of the phase change^35,40^. The melt front then begins to propagate into the material and, as shown on the right axis of Fig. 2a, proceeds rapidly to a depth of over 35 μm. Figure 2b shows the full extent of the calculated thermal data, with the color bar indicating the temperature rise at each depth and heating time.

Surface acoustic waves are confined to propagate in the near surface region with a penetration depth on the order of the wavelength. For example, for a frequency of 30 MHz, surface waves in Ti-6Al-4V will be sensitive to mechanical property changes that occur over a depth of approximately 100 μm. In Fig. 2b, the temperature rise is somewhat uniform over the near surface region, and thus it is expected that the mechanical property changes will also be relatively constant over the penetration depth, resulting in a surface acoustic wave delay that is frequency independent. The surface temperature for a significantly higher heating power density of 250 kW/cm^2^ is shown in Fig. 2c. Here, surface melting occurs at about 300 μs and there are marked thermal gradients in the near surface region within the 500 μs time window as shown in Fig. 2d. These thermal gradients can cause dispersion of surface waves since the higher frequency waves, with a shorter wavelength, will be more influenced by the near surface region while lower frequency waves will penetrate further into the cooler substrate.

This one-dimensional thermal model allows for the calculation of temperature as a function of depth and melt front position at any time after the heating laser is turned on. Next, the pulsed laser excitation and interferometric detection of the CW laser-heated surface at a given time is modeled. The material near the surface is discretized into 400 layers, with a layer thickness of 0.6 µm. The elastic properties of each layer are calculated from the mean temperature of the layer, and the elastic wave propagation problem is then reduced to an analogous problem of wave propagation in a homogeneous, isotropic layered media^41^. When surface melting occurs, the thickness of the surface layer is set as the thickness of the melt pool and the density^38^ (3920 kg/m^3^) and longitudinal wave velocity^39^ (4407 m/s) of liquid Ti-6Al-4V are used. Laser generation of ultrasound in plates^42–44^ and in multi-layer plates on a semi-infinite substrate^45^ have been previously addressed by others. The approach presented by Cheng et al. is followed in which the excitation laser source is represented as an equivalent elastic boundary source (Gaussian in space with a 10 ns pulse width), and the transfer matrix technique is used to enforce the continuity of stress and displacement across all homogeneous and isotropic layer boundaries^41^. The problem is solved in cylindrical coordinates using the integral transform technique where a Hankel transform of the elastic wave equation is taken with respect to the radial coordinate (*r*) and a Laplace transform is taken with respect to time. The normal surface displacement as a function of time at a given *r* is found through numerical inversion of the Hankel-Laplace transforms.

**Figure 3:**
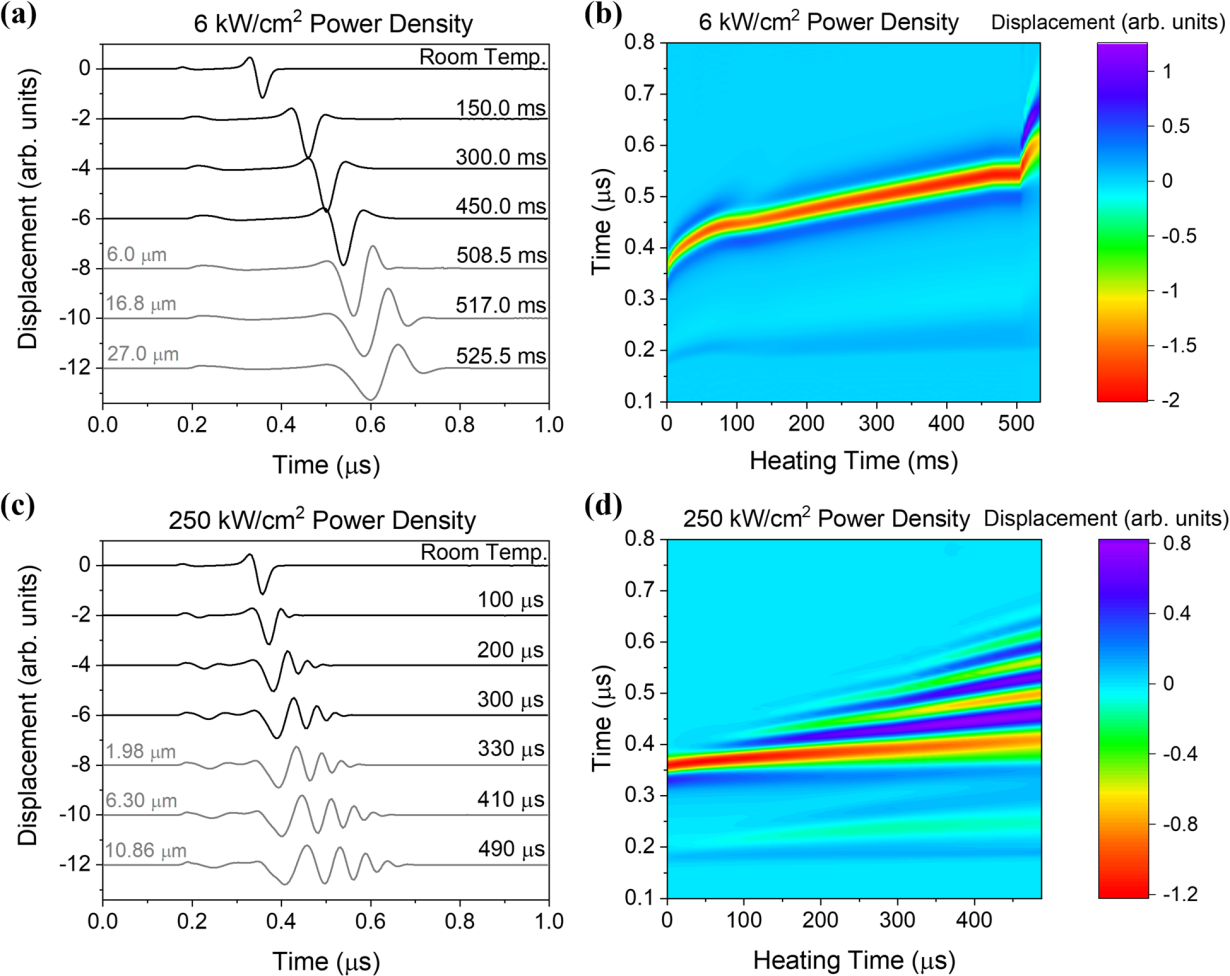
(**a**) Calculated laser ultrasonic signals with a source to detector distance of 1.0 mm at room temperature and heating times of 150.0, 300.0, 450.0, 508.5, 517.0, and 525.5 ms for a heating laser power density of 6 kW/cm^2^. (**b**) Temporal evolution of the displacement field for the 6 kW/cm^2^ power density. (**c**) Calculated laser ultrasonic signals from the 250 kW/cm^2^ heating laser power density at room temperature and heating times of 100, 200, 300, 330, 410, and 490 μs. (**d**) Temporal evolution of the displacement field for the 250 kW/cm^2^ power density.

The excitation laser spot size was set to 100 μm full width at half maximum (FWHM) with a detection location at *r* = 1.0 mm. The normal surface displacement as a function of time is given in Fig. 3a for a heating power of 6 kW/cm^2^. The top curve shows the room temperature response in the absence of CW laser surface heating. A small amplitude wave arrival corresponding to the surface skimming longitudinal wave is seen at 0.16 μs followed by the larger surface acoustic wave (SAW) amplitude arrival at about 0.30 μs. The other curves show the displacement response at various times after the CW heating laser is turned on. For the signals between *t* = 0 and *t* = 450 ms, the shape of the surface acoustic wave remains relatively uniform, but the arrival is delayed as heating proceeds. During the last three time steps: *t* = 508.5, 517.0, and 525.6 ms, melting has occurred with melt depths of 6.0, 16.8 and 27.0 μm, respectively. More prominent dispersion is seen when the surface waves traverse the molten layer; the higher frequency, short wavelength, components are delayed due to the strong interaction with the melt layer. Note that in this case, the longitudinal wave velocity in the molten liquid is higher than the shear wave velocity in the substrate so the surface waves are not leaky^46^. In general, the velocity of waves propagating on a liquid-covered half space transition from the Rayleigh wave velocity at zero thickness to the Scholte wave velocity when the thickness of the liquid is large with respect to the wavelength^47,48^.

Figure 3b shows the evolution of the displacement field calculated throughout the heating time. Here the abscissa gives the time after the heating laser is turned on while the ordinate gives the time after the excitation laser pulse. The color bar represents the normal displacement of the surface. In this image, the SAW arrival has the largest negative amplitude and is shown in red. The initial pronounced change in the SAW arrival time is associated with the rapid rise in the near surface temperature as seen in Fig. 2a,b. The SAW arrival time is then relatively constant between 75 and 100 ms during which the α → β phase transformation in Ti-6Al-4V occurs. After this transition region, the arrival of the SAW continues to be delayed with heating time in a monotonic fashion until approximately 508 ms when a sharp break in the curve associated with surface melting is observed. The SAW signal sensitivity to the presence of melt makes it an attractive option for sensing melt depth.

Figure 3c shows the normal displacement of the surface for a higher heating power density, 250 kW/cm^2^. The excitation source characteristics are the same as those given above. In this case, however, the heating takes place much more rapidly and surface melting starts at about 305 μs. Such rapid heating leads to strong near-surface thermal gradients (see Fig. 2c,d) which, in turn, lead to sharp changes in the mechanical properties within the wavelength range of the broadband surface acoustic wave. At heating times between *t* = 0 and *t* = 300 μs, a significant degree of surface acoustic wave dispersion is evident, with the higher frequency components that probe the near-surface temperature delayed with respect to the lower frequencies that penetrate further into the cooler bulk of the material. This effect is more pronounced at later times (*t* > 300 μs) where the higher frequency SAW components are also delayed by the presence of surface melt. Figure 3d shows the temporal evolution of the displacement field with surface heating. While the dispersion is certainly more pronounced than in Fig. 3b, the onset of surface melting is not as evident. Note that the dispersion of the SAWs in a multilayer system can be used to back out the depth-dependent mechanical properties using a model-based inversion approach^49^. For the heating case, depth-dependent mechanical properties could ultimately be related to the subsurface temperature profile.

## Experimental setup

**Figure 4:**
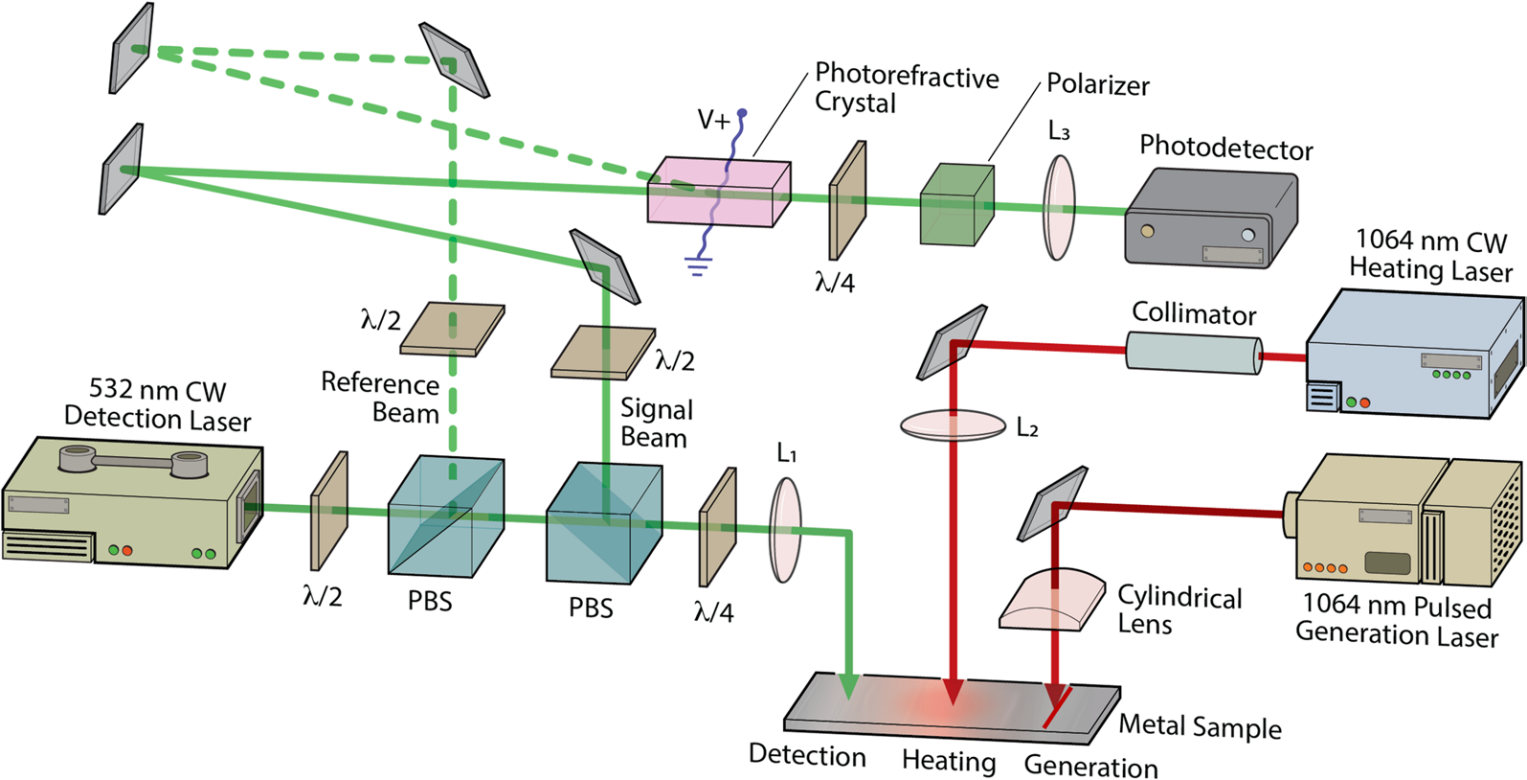
Schematic of the experimental setup depicting the three laser beams on the sample surface. The following abbreviations are used: PBS, polarizing beam splitter; λ/2, half-wave plate; λ/4, quarter-wave plate; L1, L2, L3, focusing lenses.

A laser ultrasonic system was used to study surface acoustic wave propagation through a laser-heated region of Ti-6Al-4V samples. A schematic of this experimental configuration is shown in Fig. 4. A pulsed Nd:YAG laser operating at the fundamental frequency (λ = 1064 nm) and a repetition rate of 15 Hz is used to generate broadband surface acoustic waves. The generation laser is collimated and focused to a line on the sample surface using a cylindrical lens. At the sample surface, the line source was approximately 15 mm in length and had a Gaussian FWHM of 60 μm. The laser energy at the sample was 1.6 mJ, sufficiently low that generation remained in the thermoelastic regime.

The displacement normal to the surface associated with the laser-generated acoustic wave was detected using a photorefractive crystal (PRC) based interferometer using a Bismuth Silicon Oxide (BSO) PRC^50–52^. The detection laser was a 300 mW frequency doubled Nd:YAG laser operating at 532 nm. The laser output was directed to a beamsplitter where it was divided into reference and signal beams. The reference beam was sent directly to the PRC, while the signal beam was focused onto the polished specimen surface, and, upon reflection, was sent to the PRC where it interferes with the reference beam at a 5-degree angle and creates a sinusoidal index grating inside the crystal. A portion of the reference beam diffracts from the grating in the two-wave mixing process and interferes with the signal beam at the photodetector. In addition, an AC electric field was applied across the PRC to enhance the two-wave mixing gain. Polarization optics after the PRC were used to ensure that the diffracted reference beam and transmitted signal beam were in quadrature, optimizing the detection sensitivity. The output of the photodetector was sent to a digital oscilloscope with a 200 MHz bandwidth limit and subsequently transferred to a computer and filtered using a 40 MHz second order low pass Butterworth filter. The distance between the SAW excitation line and detection point was set to 4.0 mm.

A fiber-coupled 60 W continuous wave Nd:YAG laser operating at a wavelength of 1064 nm was used to heat the sample surface. The laser output was collimated and sent through a spherical lens to the surface. The Gaussian spot size at the surface was 644 μm and the heating laser was positioned directly in between the SAW excitation laser line and the detection point using translation stages. The samples were polished Ti-6Al-4V disks with a diameter of 25 mm and a height of 13 mm. A LabVIEW code was used to control the heating laser power and to acquire laser ultrasonic signals during the heating process at a data acquisition rate of 15 Hz (corresponding to the excitation laser repetition rate). At a given heating laser power, data acquisition commenced at a heating time *t*_*h*_ = − 3 s, and single shot laser ultrasonic signals were acquired continuously throughout the experiment. At *t*_*h*_ = 0 s the heating laser was switched on and the sample was illuminated with a constant power for 10 s, after which the heating laser was turned off. To monitor sample cooling, data acquisition continued for 5 s after the heating laser was turned off. The sample was then allowed to cool to room temperature and translated to a new position. The experimental procedure was repeated a total of 10 times at each heating power and the laser ultrasonic signals collected at each time, with respect to heating laser turn on at *t*_*h*_ = 0, were averaged to improve the signal-to-noise ratio. After each experiment, the sample surface was inspected using an optical microscope for signs of surface melting and discoloration.

## Results and discussion

**Figure 5:**
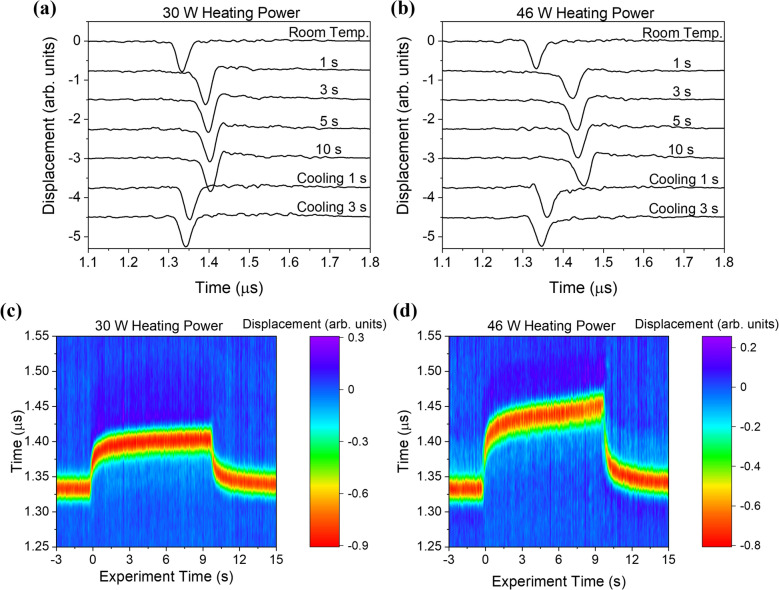
Laser ultrasonic signals in Ti-6Al-4V samples with a source to detector distance of 4.0 mm at room temperature, heating times of 1, 3, 5, and 10 s, and cooling times of 1 and 3 s for CW laser heating powers of (**a**) 30 W and (**b**) 46 W. Temporal evolution of the displacement fields, where experiment time of 0 s indicates the time at which the heating laser is turned on, for CW laser heating powers of (**c**) 30 W and (**d**) 46 W.

Figure 5a shows the ultrasonic signals detected at several heating times, with the prominent feature (negative dip) corresponding to the arrival of the surface acoustic wave. The top waveform shows the response in the absence of surface heating (*t*_*h*_ < 0) and the negative peak in the surface wave arrival is seen at about 1.33 μs. The next four curves show the displacement responses at various times after the heating laser is turned on, and the last two curves show the response at 1 and 3 s after the heating laser is turned off. The shape of the surface acoustic wave remains relatively constant, but the arrival is delayed as heating proceeds. This lack of dispersion is expected as the heating times are long and the thermal field is thus relatively constant throughout the surface wave penetration depth. Figure 5b shows similar results for a CW laser heating power of 46 W. The basic features of the waveforms at 30 W and 46 W are similar but the delay in the SAW is more pronounced with the larger heating power due to the increase in temperature in the laser-heated region.

Figure 5c,d show the evolution of the displacement field throughout these experiments for CW laser heating powers of 30 W and 46 W, respectively. The abscissa gives the experiment time, where *t*_*h*_ = 0 represents the time at which the heating laser is turned on and the heating laser is turned off at *t*_*h*_ = 10 s, while the ordinate gives the time after the excitation laser pulse. The color bar represents the out of plane displacement of the surface. In these images, the SAW arrival has the negative-most amplitude shown in red. In both images, the surface wave arrival shows a marked delay associated with the onset of surface heating. This is followed by a more gradual change between *t*_*h*_ = 2 s and *t*_*h*_ = 10 s as heat diffuses through the sample and the sample temperature begins to approach the steady state. Finally, a rapid decrease in the arrival time is observed when the heating laser is turned off. Interestingly, the 46 W heating power not only causes more of a surface wave delay but the shape of the arrival over heating time is quite different than the 30 W heating laser case, and there is not a simple linear scaling between them. Furthermore, optical microscopy revealed no visible markings left on the surface from the 30 W heating power experiments, while there were clear discolorations and surface texture changes, indicative of surface melting, resulting from the 46 W heating power experiments.

**Figure 6:**
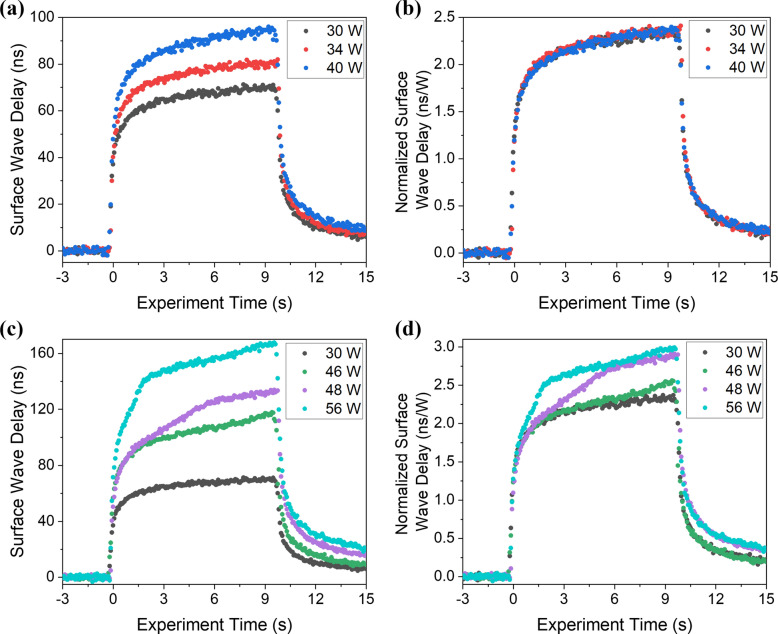
(**a**) Real-time transient surface wave delays and (**b**) normalized surface wave delays, in units of ns/W, for heating powers of 30, 34, and 40 W. (**c**) Real-time transient surface wave delays and (**d**) normalized surface wave delays for heating laser powers of 30, 46, 48, and 56 W.

The frequency content of the surface acoustic waves generated in these experiments extends to 32 MHz. At this frequency and with the slow heating time of 10 s, the SAW is minimally dispersive with the increasing temperatures as seen in Fig. 5, while the delay of the SAW is sensitive to the heating power or surface state. The arrival time of the negative peak of the surface wave was determined as a function of heating time for different laser powers. The surface wave delay was then determined by subtracting the room temperature arrival time. Figure 6a shows the real-time transient surface wave delays for heating powers of 30, 34, and 40 W. Note that at these lower heating powers, all the curves show a similar shape. If these curves are divided by the heating laser power to determine the normalized surface wave delay (in units of ns/W), all the curves collapse to a single curve as shown in Fig. 6b. This indicates that in this regime, the surface wave delay is a simple linear function of the heating power. Optical microscopy confirmed that there were no visible changes to the surface subsequent to heating in this power range. At higher heating powers the response is quite different, however: experiments performed at 46, 48, and 56 W resulted in visible markings and surface texture changes. The delay curves for these heating powers are plotted in Fig. 6c together with the results from the 30 W experiment during which no surface changes were observed. The SAW delay is significantly more pronounced and the normalized surface wave delay plots in Fig. 6d show a distinct change in shape. While all the curves follow the 30 W heating curve for early heating times, deviations begin at later times, with the deviations occurring earlier for higher power. It is hypothesized that the nonlinearity in the curve is associated with phase change and the presence of surface melt.

**Figure 7:**
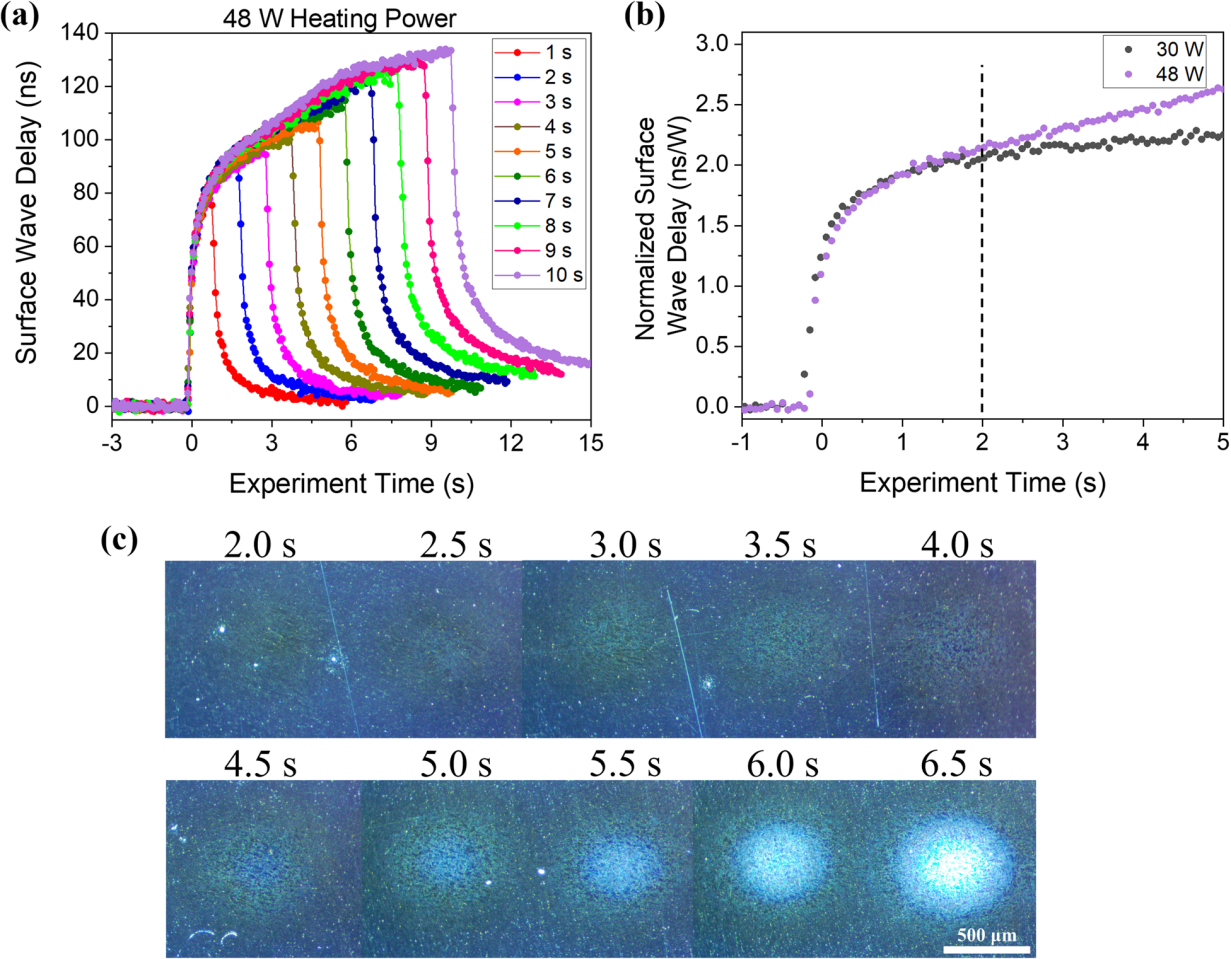
(**a**) Subset of surface wave delay data from the incremental heating experiment at a fixed laser power of 48 W. (**b**) Zoom-in of the normalized surface wave delay for heating laser powers of 30 W and 48 W. (**c**) Optical micrographs of the sample surface after the indicated times of heating.

Incremental heating experiments were performed in which the laser power was fixed at 48 W but the illumination time was varied between 0.5 s and 10.0 s in 0.5 s intervals. The sample surface was optically observed after each interval, and the sample was translated between measurements such that a new region was illuminated. Figure 7a shows a subset of the surface wave delay data, again demonstrating that surface waves can be very effective for the characterization of the transient thermal field. Figure 7b shows a zoom-in of the first 5.0 s of normalized surface wave delay for heating laser powers of 30 W and 48 W. The curves are nearly identical up until about 2.0 s of illumination, after which the 48 W curve shows a significantly higher delay. Optical micrographs of the surface are shown in Fig. 7c. There is no detectable surface discoloration before 2.0 s of heating. However, there is a clear indication of a melt region starting at 2.0 s and this region continues to grow at longer heating times.

Note that in the modeling results, a one-dimensional illumination model was used to elucidate the effects of surface heating and melting on surface wave propagation. Thus a quantitative comparison with experimental results, in which the waves propagate through a region heated with a Gaussian laser source, is not possible. Nevertheless, there is qualitative agreement between the two with a rapid delay in the surface wave velocity at the onset of illumination and further decrease in surface wave velocity associated with melting. The surface acoustic wave delay curves, such as those shown in Figs. 6 and 7, may prove useful in monitoring laser-induced thermal processes, particularly in more complex cases involving phase transformation. Changes in these curves indicate variations in the heating laser parameters or laser-material interaction. These experimental results are limited to relatively slow heating, where the temperature is uniform over the surface wave penetration depth. A higher repetition rate pulsed laser can be used to probe more rapid thermal processes and potentially obtain quantitative information about the heated zone, including temperature distribution and melt pool depth. It is also important to point out that laser additive manufacturing techniques are conducted in a layer-wise manner, and track formation and overlap add complexity to the heating and melting processes.

## Conclusion

Laser-based ultrasonics is a non-contact technique that can be used to monitor transient laser-induced heating and melting processes. Numerical simulations were performed to show that, for a spatially uniform heating beam, laser-induced surface acoustic waves are strongly influenced by surface heating conditions, are dispersive in the case of rapid heating, and that an abrupt velocity reduction happens upon the onset of surface melting. The complementary experimental results monitor the transient change of surface wave travel time associated with high power laser surface heating. Qualitative agreement between theory and experiment is observed: both show a rapid reduction in the surface wave velocity at the onset of illumination and further decrease in surface wave velocity associated with melting. This technique may ultimately find application in the mapping of transient laser-induced thermal fields and melt zones, providing critical information for real-time process control in advanced manufacturing systems including those relying on laser powder bed fusion.

## Data Availability

The datasets generated during and/or analyzed during the current study are available from the corresponding author on reasonable request.

## Appendix

See Table 1.

**Table 1:** Thermal and optical properties of Ti-6Al-4V used in the laser heating model.

Absorption depth, *α*	100 nm
Reflectivity at λ = 1064 nm, *R*	0.6154
Melting temperature, *T*_*m*_	1943 K
Vaporization temperature, *T*_*v*_	3560 K
Density, *ρ*	4.42 g/cm^3^
Atomic weight	47.897 g/mol
Latent heat of melting, *L*	390 kJ/mol
Latent heat of vaporization, *L*_*v*_	421 kJ/mol
Thermal conductivity at room temperature, *K*_*S*_	7 W/m K
Thermal conductivity (*T* < *T*_*m*_), *K*_1_	$- 0.797 + 18.2{\text{E}}{-}3T - 20{\text{E}}{-}6T^{2}$ W/m K
Thermal conductivity (*T* > *T*_*m*_), *K*_2_	34.6 W/m K
Heat capacity (*T* < *T*_*m*_),${\varvec{C}}_{{{\varvec{p}}_{1} }}$	$0.4115 + 2{\text{E}}{-}4T + 5{\text{E}}{-}10T^{2}$ J/g K
Heat capacity (*T* > *T*_*m*_),${\varvec{C}}_{{{\varvec{p}}_{2} }}$	0.83 J/g K
